# *Portulaca oleracea* L. polysaccharide inhibits ovarian cancer via inducing ACSL4-dependent ferroptosis

**DOI:** 10.18632/aging.205608

**Published:** 2024-03-19

**Authors:** Liping Xia, Mo Yang, Yan Liu

**Affiliations:** 1 Department of Ultrasound Diagnosis and Treatment, The Second Affiliated Hospital of Shandong First Medical University, Tai’an City, Shandong 271000, P.R. China

**Keywords:** *Portulaca oleracea* L. polysaccharide, ovarian cancer, ferroptosis, ACSL4

## Abstract

The antitumor effect of *Portulaca oleracea* L. polysaccharide (POL) has been demonstrated, but whether it curbs the development of ovarian cancer has not been reported. Here, we treated ovarian cancer cells with different concentrations of POL, detected cell activity by CCK-8 assay, and apoptosis rate by flow cytometry. The results showed that SKOV3 and Hey cell survival decreased with increasing POL concentration in a dose-dependent manner. POL significantly inhibited ovarian cancer cell migration and increased cell death compared with the control group. Ferroptosis inhibitors, but not apoptosis, necrosis, and autophagy inhibitors, reversed POL-induced cell death. Further studies revealed that POL promoted the accumulation of lipid reactive oxygen species (ROS), Fe2+, malondialdehyde (MDA), and decreased glutathione (GSH) production. Moreover, POL significantly increased the mortality of ovarian cancer cells. *In vivo* studies confirmed that POL reduced the volume and weight of tumors and increased the levels of Fe2+ and MDA in mice *in vivo*. Western blot assay revealed that POL increased the expression of ACSL4 in ovarian cancer cells as well as in tumors in mice *in vivo*. More importantly, the POL-mediated increase in lipid ROS, Fe2+, MDA, and decrease in GSH were significantly reversed after knocking down ACSL4 in ovarian cancer cells. Thus, POL can effectively inhibit ovarian cancer development, which may be achieved by increasing ACSL4-mediated ferroptosis. These results suggest that POL has the potential to be a potential drug for targeted treatment of ovarian cancer.

## INTRODUCTION

Ovarian cancer is one of the common malignant tumors of the female reproductive system, and the 5-year survival rate of ovarian cancer is just 30% due to the lack of early screening means, the susceptibility to chemotherapy resistance, and the high recurrence rate [1, 2]. Although surgery, chemotherapy, angiogenesis inhibitors, poly (adenosine diphosphate-ribose) polymerase (PARP) inhibitors and immunotherapy provide more options for ovarian cancer treatment, but in most cases, it is still difficult for patients to obtain satisfactory treatment results [3, 4]. Therefore, finding a newer, reliable and potent new drug is more meaningful for the treatment and prognosis of ovarian cancer [5, 6]. Ferroptosis, proposed by Dixon et al. in 2012, is a non-apoptotic form of cell death that is highly dependent on intracellular iron ions and lipid peroxides accumulation as its main feature, and can selectively kill cancer cells such as ovarian tumor cells [7]. Therefore, ferroptosis can be used as a new strategy for the diagnosis and treatment of ovarian cancer.

Ferroptosis is characterized by two major aspects: first, in terms of cellular morphology, ferroptosis leads to smaller cellular mitochondria, increased membrane density, and reduced cristae, while morphological changes in the nucleus are not obvious; second, in terms of cellular composition, ferroptosis manifests itself as an imbalance in redox homeostasis caused by an abnormal increase in iron-dependent lipid reactive oxygen species (ROS) [7–9]. Polyunsaturated fatty acids (PUFA) are important components of biological membranes and are essential for cellular life by increasing the fluidity of membrane phospholipids and maintaining cellular function [10, 11]. However, the weak carbon-hydrogen bonds between adjacent carbon-carbon double bonds of PUFA in membrane phospholipids can be oxidized by intracellular ROS via lipoxygenase to form lipid peroxides that induce ferroptosis [11, 12]. Acyl coenzyme A synthase long chain family member 4 (ACSL4) is a regulator of lipid metabolism that mediates the insertion of PUFA into membrane phospholipids for remodeling [13, 14]. Specifically, ACSL4 converts polyunsaturated fatty acids (PUFA), including arachidonic acid (AA) to AA-CoA, and then catalyzes AA-CoA to phosphatidylethanolamines (PEs) in the presence of lysophosphatidylcholine acyltransferase 3 (LPCAT3) [15, 16]. Subsequently, PEs are oxidized into PE-AA-OOH by acid-15-lipoxygenase (ALOX15) [16]. Once the cells are deficient in glutathione peroxidase 4 (GPX4)/glutathione (GSH), PE-AA-OOH undergoes a Fenton reaction with Fe^2+^, which then triggers ferroptosis [15, 16].

*Portulaca oleracea* L. is an annual herbaceous plant in the family of Portulacaceae, widely distributed in China, and is a dual-use plant [17]. Portulaca oleracea L. has been found to possess a wide range of antimicrobial, anti-inflammatory, antioxidant, and anticancer activities, and these properties are related to the pharmacological properties of the various compounds (flavonoids, alkaloids, polysaccharides, fatty acids, terpenes, etc.,) [18]. In hepatocellular carcinoma, colon cancer, and pancreatic cancer, Portulaca oleracea L. has been shown to have significant anticancer effects [19–21]. However, there is no definite conclusion as to which compound plays a major role. Unlike other compounds, Portulaca oleracea L. polysaccharide (POL) is a polymeric carbohydrate composed of several monosaccharides, which has the advantages of low toxicity and high biological activity [22]. The anti-tumor effects of POL have been demonstrated. For example, in cervical cancer, POL induced apoptosis in tumor cells by inhibiting the TLR4/NF-κB pathway [23, 24]. In addition, POL can suppress the growth of transplanted sarcoma, and its main mechanism is related to the elevated ratio of CD4+/CD8+ cells [25]. Therefore, studying the anticancer activity of POL is of great significance for expanding its application prospects.

However, whether POL inhibits the development of ovarian cancer has not been reported. In the present study, we investigated for the first time whether POL inhibits the development of ovarian cancer and its specific molecular mechanism, with a view to exploring potential drugs for the active prevention and treatment of ovarian cancer.

## MATERIALS AND METHODS

### Cell culture

Ovarian cancer cell lines SKOV3 and Hey were purchased from Punosai (https://www.procell.com.cn/, Wuhan City, China). Ovarian epithelial cells HOSE were purchased from American Type Culture Collection (ATCC, Manassas, VA, USA). The cells were cultured in RPMI-1640 medium with 10% fetal bovine serum and 1% penicillin-streptomycin in a cell culture incubator at a constant temperature of 37°C and 5% CO_2_.

### Cell-counting-kit-8 (CCK-8) assay

SKOV3, Hey and HOSE cells were cultured to logarithmic growth, digested with 0.1% EDTA-containing trypsin (Sigma-Aldrich; Merck KGaA), centrifuged, and resuspended in complete medium. After that, the concentration of suspension was counted and adjusted to 1 × 10^5^ cells/900 μl. Then, 10 μl of Portulaca oleracea L. Polysaccharide (POL, purity: 98%, Shaanxi Snout Biotechnology Co., Xi’an city, China; working solution concentration gradients were 25, 50, 100, 200, 400, 800, 1600 μg/ml) was added to the 96-well plate and 90 μl of cell suspension was added. After 48 h of incubation, the supernatant was discarded. Subsequently, 110 μl CCK-8 working solution (100 μl complete medium + 10 μl CCK-8 reagent) was added and incubated at 37°C for 30 min. Then, the OD450 was determined and the IC50 of the drug on different cells was calculated.

### 5-ethynyl-2’-deoxyuridine (EdU) cell proliferation assay

SKOV3 and Hey cells were inoculated in six-well plates at 5 × 10^5^/well and incubated at 37°C for 48 h. We diluted EdU (10 mM) with pre-warmed cell culture solution in advance, prepared a working solution of EdU at a concentration of 20 μM, and added it to the six-well plates (1 ml/well) and incubated at 37°C for 2 h. After the supernatant was discarded, the cells were fixed with 1 ml of 4% paraformaldehyde (Beijing Solarbio Science and Technology Co., Ltd.) in each well of the six-well plate for 15 min at room temperature. After three washes with PBS, 1× Hoechst 33342 working solution (Beijing Solarbio Science and Technology Co., Ltd.) was added to each well and incubated for 5 min at room temperature. Then, the cells were observed under a fluorescence microscope and counted.

### Flow cytometry assay

SKOV3 and Hey cells were inoculated in six-well plates at 5 × 10^5^/well and incubated at 37°C for 48 h. After collecting the culture fluid, we centrifuged the cell suspension at 4°C for 10 min at 1000 rpm and discarded the supernatant. Cells were resuspended with 250 μl binding buffer and the concentration was adjusted to 1 × 10^6^/ml. 100 μl of cell suspension was then incubated with 5 μl Annexin-V-PE and 10 μl 7-AAD (Annexin V-PE/7-AAD Apoptosis Detection Kit, Beijing BioLab Technology Co.) at room temperature for 15 min and subsequently analyzed by flow cytometry (CytoFLEX, Life Sciences). Data processing was performed using FlowJo TM10 software (Shanghai, China).

### Real-time quantitative PCR (RT-qPCR)

Cellular RNA was extracted by RiboPure RNA Purification Kits (Thermo Fisher), and RNA purity and concentration were determined by Nano Drop 2000 (Thermo Fisher). After that, SuperScript IV Reverse Transcription Kit (Thermo Fisher) was used to reverse transcribe the RNA into cDNA (the reaction program was 15 min at 37°C, 5s at 85°C, and 5 min at 4°C), and quantitative PCR was carried out by a 2 × SYBR Green qPCR master mix (Tarkara). qRT-PCR reaction system was (10 μl): 2 × SYBR Premix, 5 μl of SYBR Green qPCR master mix, 5 μl of SYBR Green qPCR master mix, 5 μl of SYBR Green qPCR master mix, 5 μl of SYBR Green qPCR master mix. The qRT-PCR reaction system was (10 μl): 2 × SYBR Premix, 5 μl; F-primer (10 μM), 0.4 μl; R-primer (10 μM), 0.4 μl; cDNA, 2 μl cDNA, 2 μl;dd H_2_O, 2 μl. Reaction procedures were: 95°C for 2 min, 40 cycles (95°C for 5s, 60°C for 30s), 95°C for 5s. GAPDH was used as an internal reference and the relative gene content was detected by the 2^−ΔΔCt^ method. The primer sequences were as follows:

F-ptgs2 (prostaglandin-endoperoxide synthase 2): CTGTGTCAAGCACTGTGGGT; R-ptgs2: ACACAACCCAAATTCCCAGGT; F-Chac1 (ChaC glutathione specific gamma-glutamylcyclotransferase 1): CTCAGCCCAGCCATCCATAG; R-Chac1: CAAGTGGGTAAGAGGCCCAG; F-GAPDH (glyceraldehyde-3-phosphate dehydrogenase): CCAGCAAGAGCACAAGAGGA; R-GAPDH: GGGGAGATTCAGTGTGGTGG.

### Transwell assay

Cells (2 × 10^4^ cells/100 μl) were spread in the upper chamber coated or uncoated with 50 μl of Matrigel (BD Biosciences, USA) serum-free medium. Meanwhile, RPMI-1640 containing 10% FBS was added to the lower chamber. After incubation at 37°C for 12 h, cells migrating to the bottom of the membrane were fixed with 4% paraformaldehyde (Beijing Solarbio Science and Technology Co., Ltd.) and stained with crystal violet solution for 30 min. All cells were counted under a microscope at 200× magnification.

### Scratch test

Logarithmic growth stage cells were selected and inoculated in 6-well culture plates. Then, 20 μl pipette tip was used to scratch the line vertically in the 6-well plates. PBS was used to wash away the floating cells, and complete medium containing different POL concentrations was added. After that, the pictures were taken under the microscope at 24 h. Migration rate was calculated, and all the experiments were repeated for three times.

### Lipid peroxidation

Log phase SKOV3 and Hey cells were inoculated at a density of 1 × 10^5^/cell in six-well plates at 37°C for 24 h. After the cells were adhered overnight, the cells were sequentially divided into control group and POL group for 24 h. Then C11 BODIPY 581/591 working solution (2 μM, MCE, USA) was added, and incubated at 37°C for 30 min. The cells were then photographed with a fluorescence microscope (Zeiss, Germany), and relative fluorescence intensities were calculated by ImageJ software.

### Detection of intracellular Fe^2+^ concentration

Log phase SKOV3 and Hey cells were inoculated at a density of 1 × 105/cell in six-well plates at 37°C for 24 h. After the cells were adhered overnight, the cells were sequentially divided into control group and POL group for 24 h. The cells were then continued to be incubated with 5 μM FerroOrange (MCE, USA) at 37°C for 1 h. The cells were then photographed with a fluorescence microscope (Zeiss, Germany), and relative fluorescence intensities were calculated by ImageJ software.

### Mitochondrial membrane potential (MMP) assay

1 mL of JC-1 staining working solution (Abcam, Cambridge, England) was added to 6-well plate and incubate in CO_2_ incubator at 37°C for 20 min. The cells were then photographed with a fluorescence microscope (Zeiss, Germany).

### Western blot

After 24 h of POL treatment, the cells were centrifuged at 3000 r/min for 10 min. Cells were washed twice with PBS, lysed by adding RIPA lysis solution (After 24 h of drug treatment, the cells were centrifuged at 3000 r/min for 10 min. Cells were washed twice with PBS, lysed by adding RIPA lysis solution for 30 min, and centrifuged at 12,000 r/min for 10 min at 4°C. We collected the supernatant, and protein concentration was determined by the BCA method (Thermo Fisher). 20 μl of the upper sample was taken for SDS-PAGE gel electrophoresis at 120 V for 1.5 h. After the electrophoresis, the membrane was washed by TBST and blocked with 8% non-fat milk at room temperature for 1 h. The membrane was incubated with ACSL4 (CST, USA) and GAPDH (CST, USA) primary antibody (1:1000) at 4°C overnight, and then the secondary antibody (Beijing Solarbio Science and Technology Co., Ltd., 1:5000) was added and incubated at room temperature for 1 h. The membrane was washed with TBST, and the intensity was developed by ECL method. The relative expression of target protein was analyzed by ImageJ software, and the relative expression of target protein was represented by the gray value of target protein bands/GAPDH bands.

### Determination of reduced glutathione (GSH) and malondialdehyde (MDA)

Cells were inoculated into 6-well plates and collected after full growth. The intracellular GSH and MDA levels were determined using the reduced glutathione (GSH) assay kit (Beijing Solarbio Science and Technology Co., Ltd.) and malondialdehyde (MDA) assay kit (Beijing Solarbio Science and Technology Co., Ltd.). The experimental procedures were carried out in strict accordance with the instructions.

### Nude mice tumor assay

Nude mice (Sipeifu biotech, Beijing, China) were acclimatized and fed for one week to prevent stress caused by changes in the environment and feeding conditions. SKOV3 cells were collected and the concentration was adjusted to 5 × 10^8^/ml. Nude mice were induced by ether anesthesia, and then anesthetized by intraperitoneal injection of 1% pentobarbital sodium solution at a dose of 10 μl/g body weight. After the nude mice were completely anesthetized, the abdominal wall was disinfected and 10 μl of cell suspension was injected with a micro syringe. Two weeks later, all the nude mice were randomly grouped into the PBS group and the POL group, and 100 μl of PBS or POL was injected twice a week at a dose of 50 mg/kg for 3 weeks. During this period, the nude mice were closely observed in terms of their appetites, spirits and body weights. After the drug administration, the mice were asphyxiated by CO_2_, some fresh tissue samples were directly embedded by OCT, and some other tissues were cut into small pieces and stored at −80°C in a low-temperature refrigerator.

### Immunohistochemistry (IHC)

Tumor tissues obtained from previous animal experiments were fixed with 4% paraformaldehyde. After further dewaxing and dehydration for antigen repair, washing, sealing, the slides were incubated with anti-ACSL4 antibody (1:100) at 4°C overnight. The sections were rinsed 3 times with 1× PBS slowly, and the tissue on the sections was incubated with the secondary antibody for 15 min followed by prepared DAB color development solution at room temperature for 5 min. Subsequently, the blocks were re-stained, differentiated, reversed blue, dehydrated and transparent. Finally, a small amount of neutral resin was added and the coverslips were sealed. And representative images were taken with a Nikon DS-Ri2 microscope.

### TdT-mediated dUTP nick end labeling (TUNEL)

Tumor tissues obtained from previous animal experiments were fixed with 4% paraformaldehyde. After further dewaxing and dehydration for antigen repair, washing, sealing, sections were blocked with PBS containing 5% goat serum (BSA) for 1 h at room temperature, followed by three rinses with TBST at room temperature. The Enzyme Solution and Label Solution were mixed 1:10 and kept away from light. A drop of 20 ul mixture was added to each slide and incubated for 10 min at room temperature away from light and rinsed three times with PBS at room temperature. Subsequently, 10 μl of DAPI staining solution was added to each slide and incubated at room temperature for 5 min. After rinsing with PBS for three times at room temperature, the slides were sealed with sealing agent, and the results were observed under a fluorescence microscope. By image pro software, the positive points of DAPI were counted as the total number of cells, and the positive points of TUNEL were counted as the tunel points (TUNEL/DAPI × 100% as the ratio of apoptotic cells).

### Statistical analysis

All the data were analyzed and graphed using GraphPad Prism software. The experimental data are presented as the mean ± SD. Two-group comparisons were analyzed by Student’s two-sided *t*-test, and multiple group comparisons were analyzed by one-way ANOVA + Tukey’s two-sided test. *P* < 0.05 was statistically significant.

## RESULTS

### POL reduces ovarian cancer cell proliferation in a dose-dependent manner

**Figure 1:**
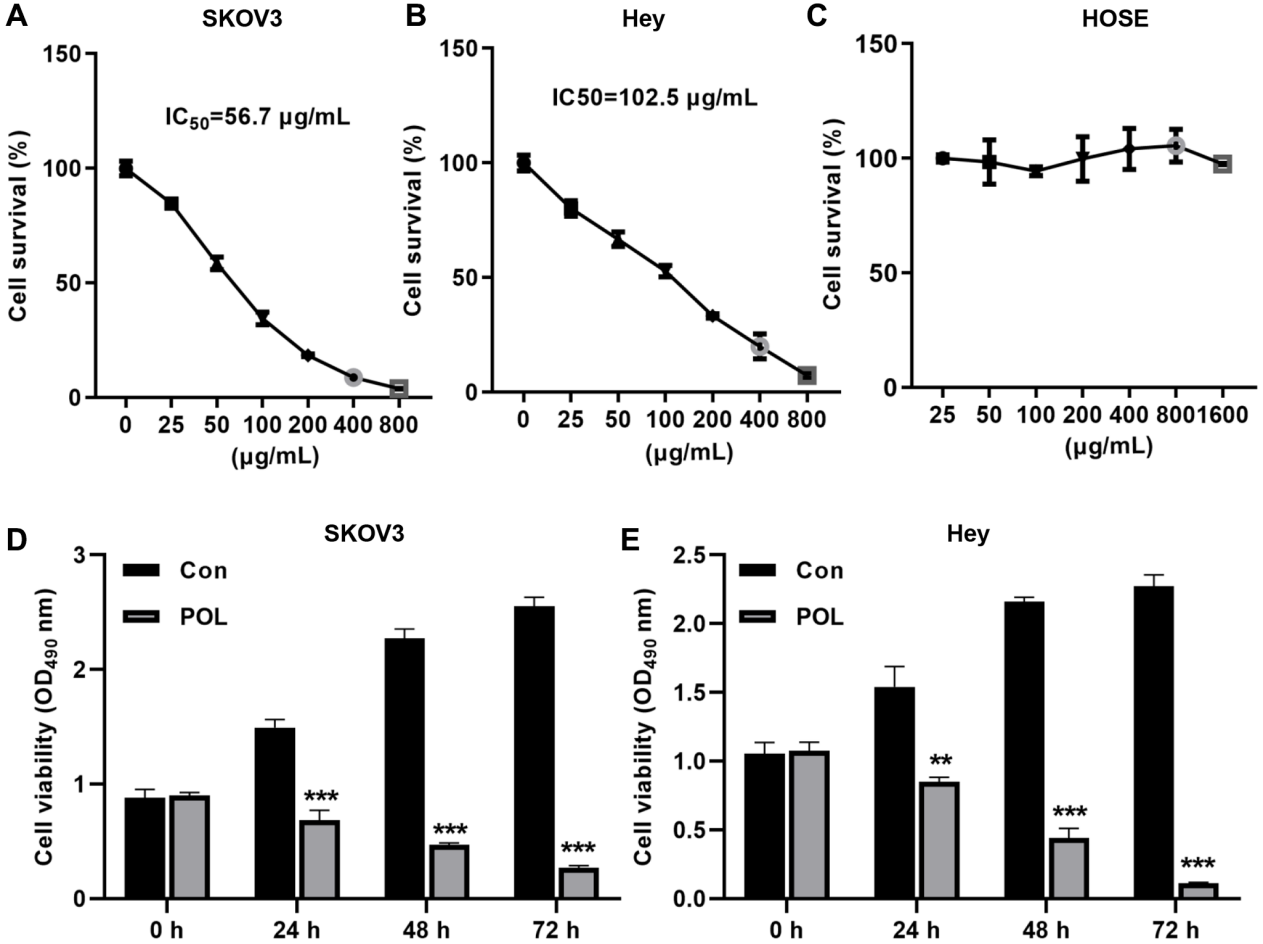
**POL reduced the proliferation of ovarian cancer cells in a dose-dependent manner.** CCK-8 was assayed whether 25, 50, 100, 200, 400, 800, 1600 μg/ml of POL reduced the cell viability of SKOV3 (**A**), Hey (**B**) as well as HOSE (**C**). At 24 h, 48 h, and 72 h, 50 and 100 μg/ml of POL attenuated the viability of SKOV3 (**D**) and Hey (**E**) cells. ^**^P < 0.01; ^***^P < 0.001 vs. Con.

First, we examined the effect of POL on the malignant proliferation of ovarian cancer cells. As shown in Figure 1A, 1B, POL decreased the viability of SKOV3 and Hey cells in a dose-dependent manner. Among them, in SKOV3 cells, the IC50 was 56.7 μg/ml; while in Hey cells, the IC50 was 102.5 μg/ml. In the next experiments, we chose 50 and 100 μg/ml of POL to treat SKOV3 and Hey cells, respectively. We also tested whether the same concentration of POL affected the proliferation of HOSE in mammary epidermal cells. CCK-8 assay revealed that the same concentration of POL had almost no effect on the proliferation of HOSE (Figure 1C). Moreover, POL at 24 h, 48 h, 72 h, 50 and 100 μg/ml attenuated the viability of SKOV3 and Hey cells and showed a time gradient dependence (Figure 1D, 1E).

### POL inhibits migration and induces cell death in ovarian cancer cells

**Figure 2:**
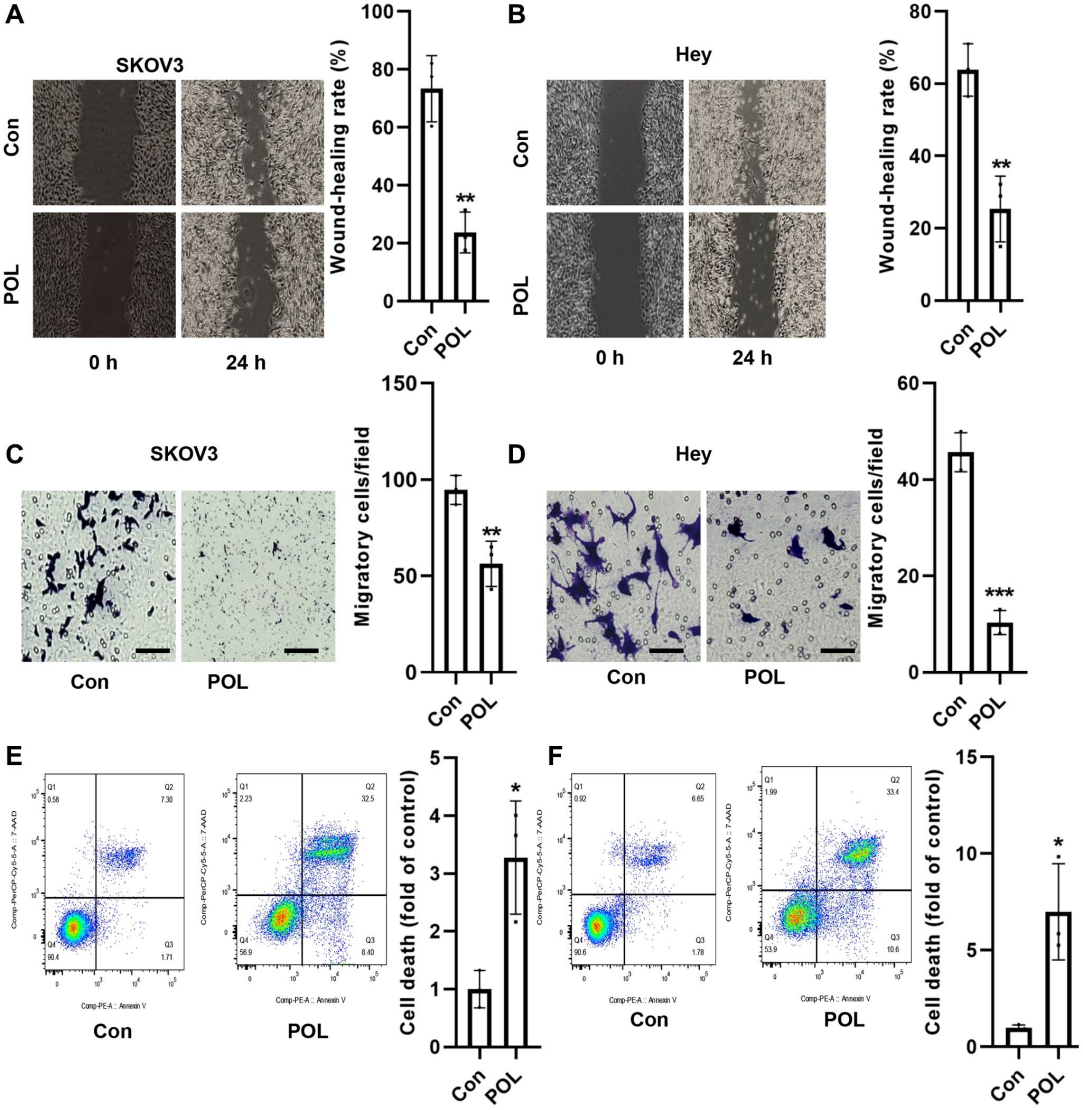
**POL inhibits migration and induces cell death in ovarian cancer cells.** Scratch assay revealed that 50 and 100 μg/ml of POL significantly inhibited the migration of SKOV3 (**A**) and Hey (**B**) cells at 48 h (4×). Transwell assay revealed that POL effectively inhibited the migration of SKOV3 (**C**) and Hey (**D**) cells (20×). Flow cytometry results assay revealed that 50 and 100 μg/ml of POL significantly increased the mortality of SKOV3 (**E**) and Hey (**F**) cells. ^*^P < 0.05, ^**^P < 0.01, ^***^P < 0.001 vs. Con.

Scratch assay revealed that 50 and 100 μg/ml of POL significantly inhibited the migration of SKOV3 and Hey cells at 48 h (Figure 2A, 2B). Similarly, transwell assay also confirmed that POL could effectively inhibit the migration rate of SKOV3 and Hey cells (Figure 2C, 2D). Flow cytometry results revealed that 50 and 100 μg/ml of POL significantly increased the mortality of SKOV3 and Hey cells (Figure 2E, 2F). These results suggested us that POL could effectively improve the malignant proliferative phenotype of ovarian cancer *in vitro*.

### POL induces ferroptosis in SKOV3 and Hey cells

**Figure 3:**
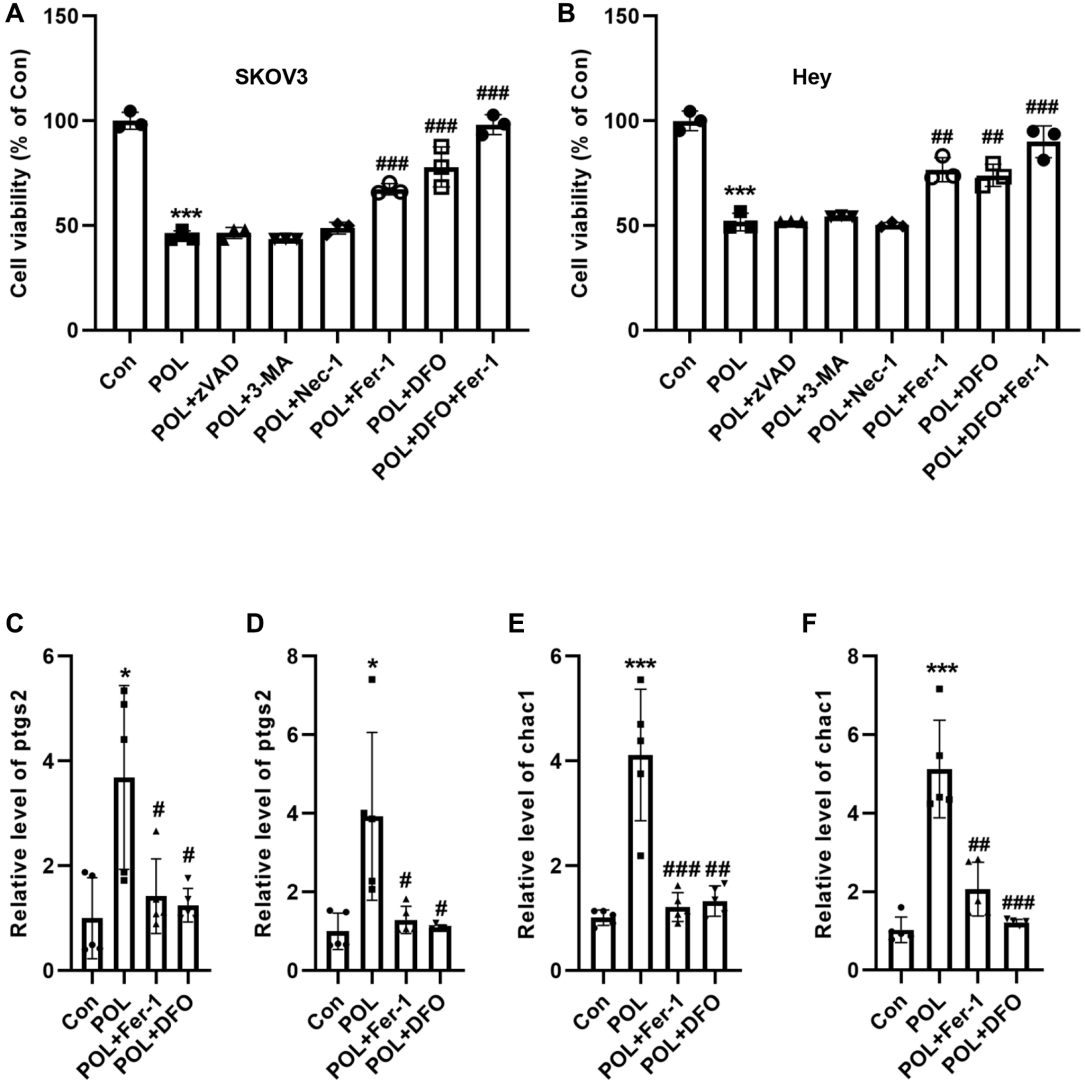
**POL induced ferroptosis in SKOV3 and hey cells.** CCK-8 assay showed that ferroptosis inhibitor, Fer-1 and DFO, were able to reverse the POL-induced decrease in cell viability of SKOV3 (**A**) and Hey (**B**) cells. RT-PCR analysis showed that POL was able to significantly increase the mRNA levels of ptgs2 and Chac1 in SKOV3 (**C**, **D**) and Hey (**E**, **F**) cells. ^*^P < 0.05, ^***^p < 0.001 vs. Con; ^#^*P* < 0.05, ^##^*P* < 0.01, ^###^*P* < 0.001 vs. POL.

Next, we analyzed by which way POL induced SKOV3 and Hey cell death. W SKOV3 and Hey cells were pre-incubated with an apoptosis inhibitor (Z-VAD-FMK, 20 μM), an autophagy inhibitor (3-methyladenine, 3-MA, 20 μM), a necrosis inhibitor (Necrostatin-1, Nec-1, 10 μM), and a Ferroptosis inhibitor (Ferrostatin-1 (Fer-1) and Deferoxamine (DFO), 1 μM) for 1 h, followed by continued co-incubation with POL for 24 h. As a result, it was found that only Fer-1 and DFO were able to reverse POL-induced decrease in ovarian cell viability (Figure 3A, 3B). Specifically, in SKOV3 and Hey cells, POL reduced the viability of SKOV3 and Hey cells to 45% and 50%, respectively, compared with the control. In contrast, Fer-1 (a lipid ROS scavenger) and DFO (an iron chelator) elevated SKOV3 and Hey cell viability to 67%, 77% and 78%, 74%, respectively. When the two were combined, SKOV3 and Hey cell viability increased to 98% and 90%, respectively (Figure 3A, 3B), suggesting that POL-induced ferroptosis is driven by iron-dependent lipid peroxidation. We further explored the effects of POL on two markers of ferroptosis, ptgs2 and Chac1. POL was able to significantly increase the mRNA levels of ptgs2 and Chac1 in SKOV3 and Hey cells (Figure 3C–3F). These results suggest that ferroptosis plays a dominant role in POL-induced cell death in ovarian cancer.

### POL increases ACSL4 expression in a concentration gradient-dependent manner

**Figure 4:**
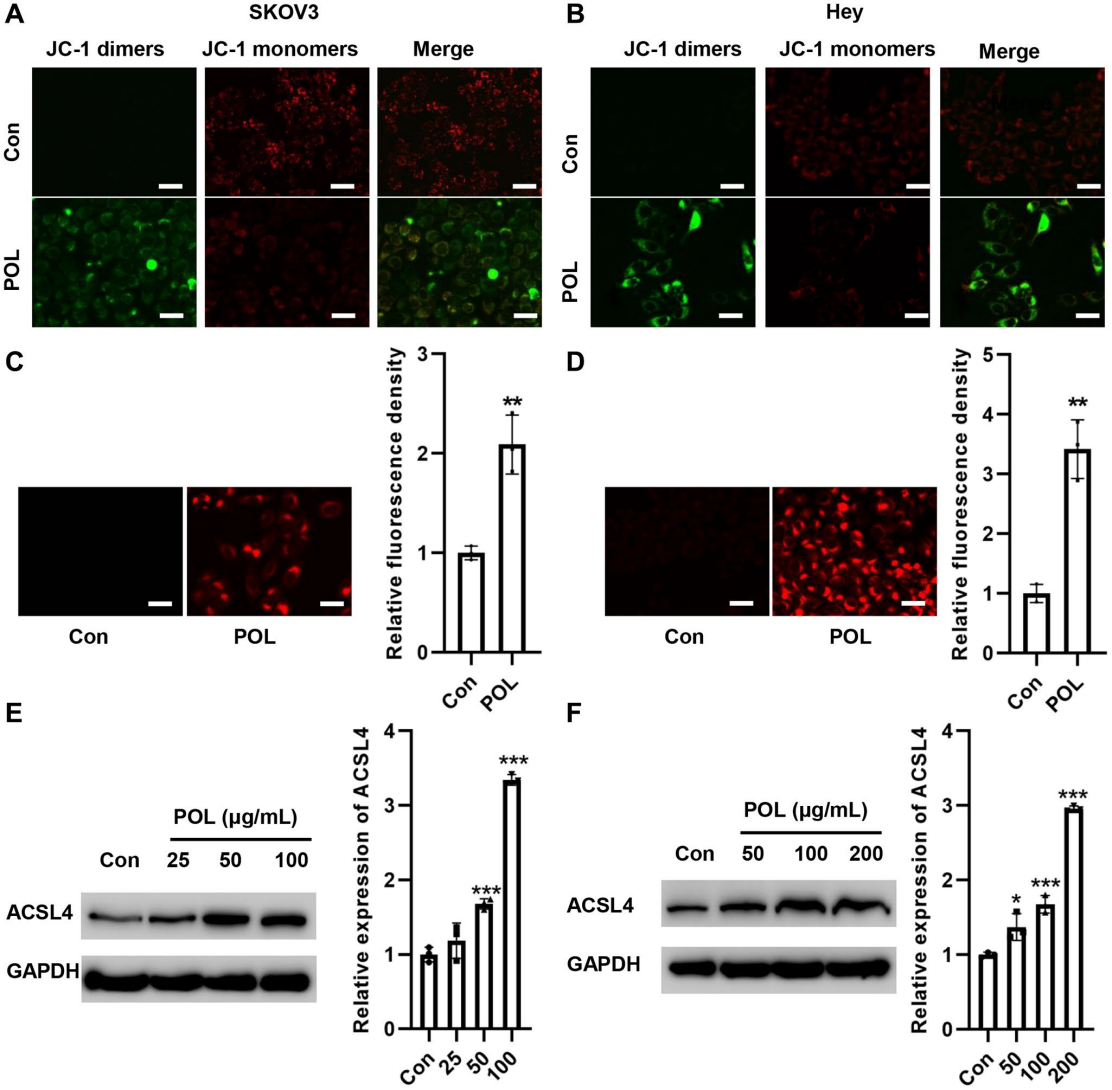
**POL increased ACSL4 expression in a concentration gradient-dependent manner.** JC-1 analysis showed that POL decreased MMP in SKOV3 (**A**) and Hey (**B**) cells (20×). FerroOrange staining showed that POL increased Fe^2+^ levels in SKOV3 (**C**) and Hey (**D**) cells (20×). Western blot assay revealed that POL increased the protein expression level of ACSL4 in SKOV3 (**E**) and Hey (**F**) in a concentration gradient-dependent manner. ^**^P < 0.01, ^***^p < 0.001 vs. Con.

We examined the effect of POL on MMP. As shown in Figure 4A, 4B, JC-1 was mainly present as JC-1 monomers in the control group, whereas after POL treatment, JC-1 was mainly present as JC-1 dimers, suggesting that POL induced a decrease in mitochondrial MMP, which led to mitochondrial dysfunction and triggered ferroptosis (Figure 4A, 4B). Meanwhile, POL significantly increased Fe^2+^ levels in SKOV3 and Hey cells (Figure 4C, 4D). We further detected the changes in ferroptosis-related protein expression. The results, as shown in Figure 4E, 4F, showed that POL increased the protein expression level of ACSL4 in a concentration gradient-dependent manner.

### Knockdown of ACSL4 reverses POL-induced ferroptosis in ovarian cancer cells

**Figure 5:**
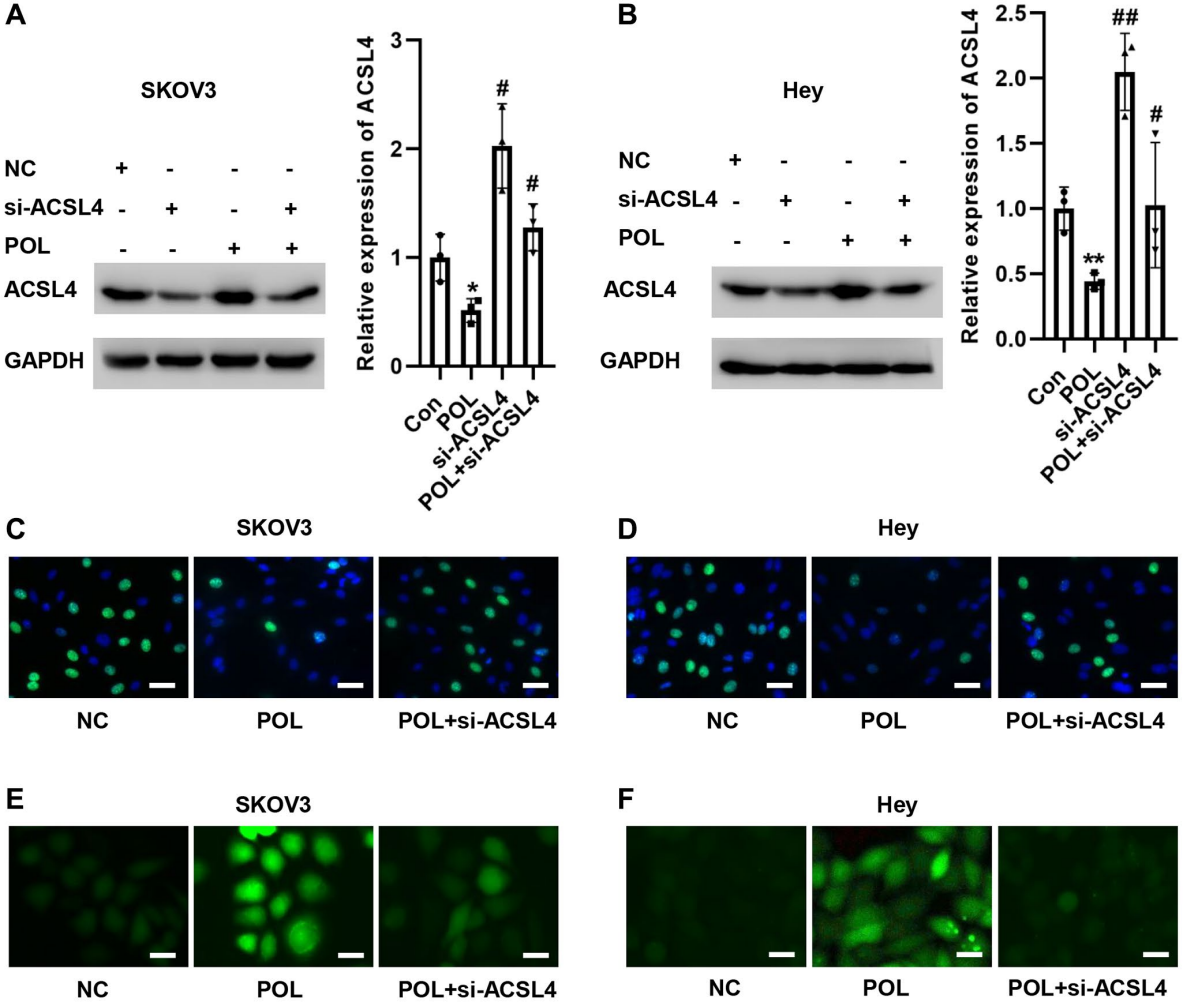
**Knockdown of ACSL4 reverses POL-induced ferroptosis in ovarian cancer cells.** Western blot assay revealed that POL-induced high expression of ACSL4 was reversed to some extent by si-ACSL4 in SKOV3 (**A**) and Hey (**B**) cells. EdU staining showed that POL decreased the proliferation of SKOV3 (**C**) and Hey (**D**) cells, and si-ACSL4 could reverse this effect (20×). Silencing ACSL4 also reduced POL-induced lipid peroxidation in SKOV3 (**E**) and Hey (**F**) cells (40×). ^*^P < 0.05, ^**^p < 0.01 vs. Con; ^#^*P* < 0.05, ^##^*P* < 0.01 vs. POL.

Next, we screened siRNAs specifically targeting ACSL4. si-ACSL4 effectively suppressed ACSL4 expression in SKOV3 and Hey cells as shown in Figure 5A, 5B. Meanwhile, POL-induced high ACSL4 expression was reversed to some extent by si-ACSL4 (Figure 5A, 5B). In addition, we examined the effect of POL on cell proliferation by EdU staining. Compared with the control group, POL reduced the proliferation of SKOV3 and Hey cells, and si-ACSL4 could reverse this effect (Figure 5C, 5D). Also, silencing ACSL4 reduced POL-induced lipid peroxidation in SKOV3 and Hey cells (Figure 5E, 5F). This result confirms that ACSL4 plays a major role in POL-mediated ferroptosis in ovarian cancer cells.

### POL inhibits tumor growth in mice *in vivo*

**Figure 6:**
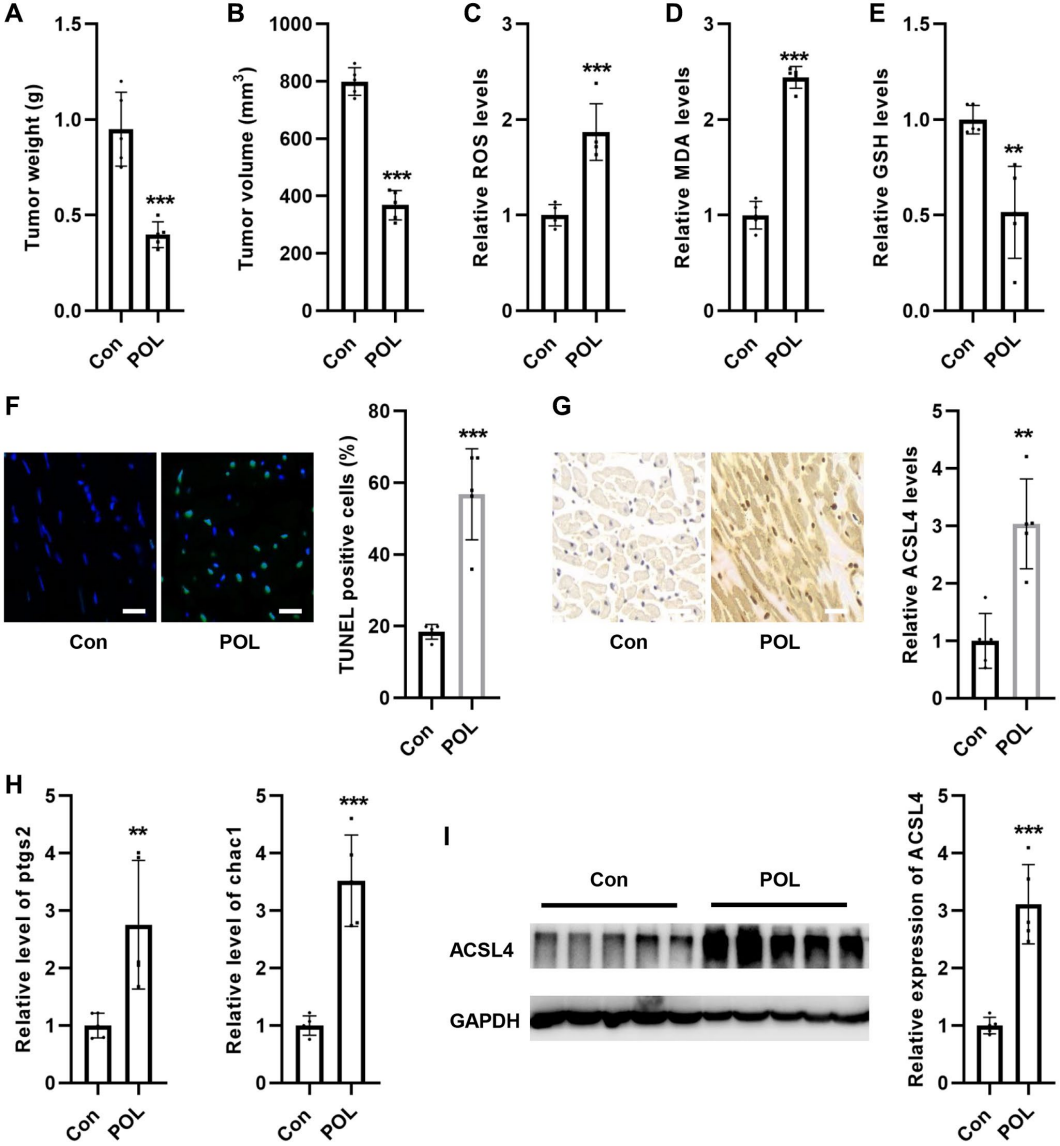
**POL inhibited tumor growth in mice *in vivo*.** POL significantly reduced the weight (**A**) and volume (**B**) of mouse tumors. POL increased the levels of ROS (**C**) and MDA (**D**) in mouse tumor tissues. (**E**) POL decreased GSH levels in mouse tumor tissues. (**F**) TUNEL staining revealed that POL increased the cell death rate in tumor tissues (20×). (**G**) IHC staining revealed that POL increased the level of ACSL4 in tumor tissues (20×). (**H**) RT-PCR results revealed that POL increased the mRNA levels of ptgs2 as well as chac1 in mouse tumor tissues. (**I**) Western blot similarly confirmed that POL increased the protein expression level of ACSL4 in mouse tumor tissues. ^**^P < 0.01, ^***^p < 0.001 vs. Con.

Next, we explored the effect of POL on tumor volume in mice *in vivo*. As shown in Figure 6A, 6B, POL significantly reduced tumor weight and volume. At the same time, POL increased the levels of ROS and MDA in mouse tumor tissues (Figure 6C, 6D). Conversely, POL decreased GSH levels in mouse tumor tissues (Figure 6E). TUNEL staining revealed that POL increased cellular mortality in tumor tissues (Figure 6F). IHC staining revealed that POL increased the levels of ACSL4 in tumor tissues (Figure 6G). RT-PCR results revealed that POL increased mouse tumor mRNA levels of ptgs2 as well as chac1 in the tissues (Figure 6H). Western blot similarly confirmed that POL increased the protein expression levels of ACSL4 in mouse tumor tissues (Figure 6I). These results suggest that POL is an effective cancer inhibitor.

## DISCUSSION

Ovarian cancer is the malignant tumor with the highest mortality rate of the female reproductive system worldwide [26, 27]. Most women delay diagnosis due to the lack of typical clinical symptoms in the early stage, and 75% of the cases are already advanced when diagnosed [28]. Currently, the mainstay of treatment for ovarian cancer consists of surgery and platinum-based chemotherapy [28]. Even if patients initially respond well to this treatment, 70% of cases will recur after treatment, and the 5-year survival rate of patients is only 20% to 30% [28, 29]. Therefore, the identification of key biotherapeutic targets and precise mechanisms of action can provide an effective treatment strategy to prolong the survival of ovarian cancer patients.

POL plays an extremely important role in life activities by lowering body glucose, eliminating body inflammation, changing intestinal morphology, improving growth performance, and antitumor effects [30]. Yi S et al. found that Portulaca oleracea L. extract maintains the balance of intestinal flora by inhibiting Wnt/β-catenin signaling, which in turn inhibits colon cancer [31]. In pancreatic cancer, Portulaca oleracea L. extract promotes apoptosis by increasing the expression of p53 and decreasing the protein level of cyclin-dependent kinases [21]. In the present study, it was found that different doses of POL could significantly increase the proliferation inhibition rate, inhibit the migration of ovarian cancer cells and promote apoptosis. The results of this study suggest that POL may exert anti-ovarian cancer effects by inhibiting cell proliferation and inducing apoptosis. Next, we explored which form of death is primarily induced by POL. CCK-8 analysis revealed that ferroptosis inhibitors, but not apoptosis, necrosis, or autophagy inhibitors, reversed POL-induced decrease in cell viability. Meanwhile, POL significantly increased the expression of ferroptosis markers, ptgs2 and Chac1, in ovarian cancer cells, indicating that POL induced ferroptosis. In Hela and HepG2 cells, POL can cause cell cycle arrest and inhibit malignant proliferation of tumor cells [32]. Also, POL inhibits the activation of TLR4/NF-κB signaling in Hela cells, which induces apoptosis [23]. Similar to previous studies, we found that POL could block the cell cycle in S phase, and it could hinder the malignant proliferation of ovarian cancer cells. Differently, we found that apoptosis inhibitors did not reverse the POL-induced decrease in ovarian cancer cell viability. In contrast, two inhibitors of ferroptosis ameliorated POL-induced cell death, suggesting that ferroptosis, but not apoptosis, is the main cause of POL inhibition in ovarian cancer.

Ferroptosis is an important form of programmed cell death that can be induced by modulating various endogenous components, such as increasing levels of iron, glutamate, phospholipids, and PUFA or depleting GSH, GPX4, and lipid antioxidants [33, 34]. Increasing evidence suggests that ferroptosis inducers have anticancer potential [33, 34]. Therefore, exploring effective ferroptosis inducers provides new options for future treatment of ovarian cancer by inducing cellular ferroptosis. So, by what mechanism does POL cause ferroptosis in ovarian cancer cells? ACSL4 is a member of the long-chain lipoyl CoA synthetase family and catalyzes the synthesis of lipoyl CoA in fatty acid catabolism [35]. ACSL4 has been found to be a key gene in the ferroptosis pathway. ACSL4 synthesizes arachidonic acid and adrenic acid into arachidonoyl CoA and adrenoyl CoA, respectively, to participate in membrane phospholipid synthesis. Under the treatment of ferroptosis inducers such as RSL3, long-chain polyunsaturated fatty acids in the membrane are easily oxidized, which triggers ferroptosis. Thus, ACSL4 is an essential molecule for the onset of ferroptosis by promoting the oxidation of membrane phospholipids [36]. In mouse embryonic fibroblast (MEF) cells, knockdown of ACSL4 increased cellular resistance to RSL3-mediated death, an inducer of ferroptosis [37]. In glioma cells, knockdown of ACSL4 inhibited glioma cell ferroptosis and promoted tumor growth [35]. Therefore, ACSL4 is an important factor in ferroptosis and can be used as a potential therapeutic target to stimulate ferroptotic cell death. We found for the first time that POL could promote ACSL4 expression in SKOV3 and Hey cells. The *in vivo* results likewise confirmed that POL significantly inhibited tumor growth and increased MDA/ROS/Fe2+ levels and decreased GSH levels in tumor tissues. Moreover, in tumor tissues, POL similarly inhibited the expression of ACSL4.

ACSL4 is a key enzyme that catalyzes the conversion of PUFA to fatty acyl-CoA esters, which is subsequently converted by LPCAT3 to AA-PE and thus doped into phospholipid membranes [38]. AA-PE in the cell membrane can be converted to AA-OOH-PE by lipoxygenases (LOXs), and when the GPX4/Xc system (consisting of the transporter subunit SLC7A11 and the regulatory subunit SLC3A2) is inactivated, the iron oxidative defense mechanism is disrupted, leading to iron-dependent cell death known as ferroptosis [39]. In the present study, we found that two inhibitors of ferroptosis, Fer-1 (a lipid ROS scavenger) and DFO (an iron chelator), both alleviated POL-induced ferroptosis, suggesting that in ovarian cancer, the mechanism of POL-induced ferroptosis is iron-dependent lipid peroxidation.

It has been reported that the absence of ACSL4 results in the inability of long-chain polyunsaturated fatty acids in biofilm phospholipids to generate peroxides, and therefore, a large amount of intracellular lipid oxides will not accumulate in the cell to cause ferroptosis in the presence of ferroptosis-inducing agents [12, 40]. The experimental results also showed that once the expression of ACSL4 was knocked down in SKOV3 and Hey cells, the POL-induced increase in MDA/Fe^2+^/ROS, as well as the decrease in GSH, could be significantly reversed. These results suggest that POL-induced ferroptosis in ovarian cancer cells is mainly mediated by increasing ACSL4 expression.

However, there are some limitations in this study. First, we did not analyze whether other related proteins are involved in POL-induced ferroptosis. Second, it remains to be further determined whether any of its molecules upstream of ACSL4 are involved in the POL-regulated ferroptosis pathway. Third, one of the major components of cell membranes or membrane-bound organelles is lipids, and lipid peroxidation in cell membranes can impair membrane structure and function [41]. ACSL4 and LPCAT3 mainly esterify PUFA thereby encapsulating it into membrane phospholipids, which in turn serve as substrates for lipid peroxidation by lipoxygenases (ALOX5, ALOX12, ALOX12B, ALOX15, ALOX15B, ALOXE3) [41]. In the presence of iron overload, the Fenton reaction is initiated with lipid hydroperoxide, causing ferroptosis [41]. The upregulation of ALOXs is inextricably linked to ferroptosis [42, 43]. In the placenta of women with a first episode of psychosis during pregnancy, ALOX5 expression is significantly elevated along with increased iron deposition [42]. During myocardial ischemia-reperfusion injury, overexpression of ALOX15 caused cardiomyocyte ferroptosis [43]. Therefore, as a next step, we should deeply explore whether POL affects the expression of LPCAT3 and ALOXs.

Taken together, we conclude that POL inhibits ovarian cancer development by inducing ferroptosis in ovarian cancer cells through up-regulation of ACSL4. This study reveals for the first time the major molecular mechanism of ovarian cancer inhibition by POL and lays the foundation for the discovery of potential targets for ovarian cancer therapy.

## References

[1] Zhang R, Siu MKY, Ngan HYS, Chan KKL. Molecular Biomarkers for the Early Detection of Ovarian Cancer. Int J Mol Sci. 2022; 23:12041. 10.3390/ijms231912041 PMC9569881 36233339

[2] Cai L, Hu X, Ye L, Bai P, Jie Y, Shu K. Long non-coding RNA ADAMTS9-AS1 attenuates ferroptosis by Targeting microRNA-587/solute carrier family 7 member 11 axis in epithelial ovarian cancer. Bioengineered. 2022; 13:8226–39. 10.1080/21655979.2022.2049470 35311457 PMC9161843

[3] Cang W, Wu A, Gu L, Wang W, Tian Q, Zheng Z, Qiu L. Erastin enhances metastatic potential of ferroptosis-resistant ovarian cancer cells by M2 polarization through STAT3/IL-8 axis. Int Immunopharmacol. 2022; 113:109422. 10.1016/j.intimp.2022.109422 36410184

[4] Carbone M, Melino G. Stearoyl CoA Desaturase Regulates Ferroptosis in Ovarian Cancer Offering New Therapeutic Perspectives. Cancer Res. 2019; 79:5149–50. 10.1158/0008-5472.CAN-19-2453 31615810

[5] Chen Y, Liao X, Jing P, Hu L, Yang Z, Yao Y, Liao C, Zhang S. Linoleic Acid-Glucosamine Hybrid for Endogenous Iron-Activated Ferroptosis Therapy in High-Grade Serous Ovarian Cancer. Mol Pharm. 2022; 19:3187–98. 10.1021/acs.molpharmaceut.2c00333 35939328

[6] Cheng Q, Bao L, Li M, Chang K, Yi X. Erastin synergizes with cisplatin via ferroptosis to inhibit ovarian cancer growth in vitro and in vivo. J Obstet Gynaecol Res. 2021; 47:2481–91. 10.1111/jog.14779 33882617

[7] Dixon SJ, Lemberg KM, Lamprecht MR, Skouta R, Zaitsev EM, Gleason CE, Patel DN, Bauer AJ, Cantley AM, Yang WS, Morrison B 3rd, Stockwell BR. Ferroptosis: an iron-dependent form of nonapoptotic cell death. Cell. 2012; 149:1060–72. 10.1016/j.cell.2012.03.042 22632970 PMC3367386

[8] Dong H, He L, Sun Q, Zhan J, Li J, Xiong X, Zhuang L, Wu S, Li Y, Yin C, He Q. Inhibit ALDH3A2 reduce ovarian cancer cells survival via elevating ferroptosis sensitivity. Gene. 2023; 876:147515. 10.1016/j.gene.2023.147515 37247796

[9] Feng S, Yin H, Zhang K, Shan M, Ji X, Luo S, Shen Y. Integrated clinical characteristics and omics analysis identifies a ferroptosis and iron-metabolism-related lncRNA signature for predicting prognosis and therapeutic responses in ovarian cancer. J Ovarian Res. 2022; 15:10. 10.1186/s13048-022-00944-y 35057848 PMC8772079

[10] Yamada N, Karasawa T, Kimura H, Watanabe S, Komada T, Kamata R, Sampilvanjil A, Ito J, Nakagawa K, Kuwata H, Hara S, Mizuta K, Sakuma Y, et al. Ferroptosis driven by radical oxidation of n-6 polyunsaturated fatty acids mediates acetaminophen-induced acute liver failure. Cell Death Dis. 2020; 11:144. 10.1038/s41419-020-2334-2 32094346 PMC7039960

[11] Phadnis VV, Snider J, Varadharajan V, Ramachandiran I, Deik AA, Lai ZW, Kunchok T, Eaton EN, Sebastiany C, Lyakisheva A, Vaccaro KD, Allen J, Yao Z, et al. MMD collaborates with ACSL4 and MBOAT7 to promote polyunsaturated phosphatidylinositol remodeling and susceptibility to ferroptosis. Cell Rep. 2023; 42:113023. 10.1016/j.celrep.2023.113023 37691145 PMC10591818

[12] Merkel M, Goebel B, Boll M, Adhikari A, Maurer V, Steinhilber D, Culmsee C. Mitochondrial Reactive Oxygen Species Formation Determines ACSL4/LPCAT2-Mediated Ferroptosis. Antioxidants (Basel). 2023; 12:1590. 10.3390/antiox12081590 PMC10451816 37627584

[13] Zhang HL, Hu BX, Li ZL, Du T, Shan JL, Ye ZP, Peng XD, Li X, Huang Y, Zhu XY, Chen YH, Feng GK, Yang D, et al. PKCβII phosphorylates ACSL4 to amplify lipid peroxidation to induce ferroptosis. Nat Cell Biol. 2022; 24:88–98. 10.1038/s41556-021-00818-3 35027735

[14] Ding K, Liu C, Li L, Yang M, Jiang N, Luo S, Sun L. Acyl-CoA synthase ACSL4: an essential target in ferroptosis and fatty acid metabolism. Chin Med J (Engl). 2023; 136:2521–37. 10.1097/CM9.0000000000002533 37442770 PMC10617883

[15] Liao P, Wang W, Wang W, Kryczek I, Li X, Bian Y, Sell A, Wei S, Grove S, Johnson JK, Kennedy PD, Gijón M, Shah YM, Zou W. CD8^+^ T cells and fatty acids orchestrate tumor ferroptosis and immunity via ACSL4. Cancer Cell. 2022; 40:365–78.e6. 10.1016/j.ccell.2022.02.003 35216678 PMC9007863

[16] Lee H, Gan B. Ferroptosis execution: Is it all about ACSL4? Cell Chem Biol. 2022; 29:1363–5. 10.1016/j.chembiol.2022.08.002 36113403

[17] Lv WJ, Huang JY, Li SP, Gong XP, Sun JB, Mao W, Guo SN. *Portulaca oleracea* L. extracts alleviate 2,4-dinitrochlorobenzene-induced atopic dermatitis in mice. Front Nutr. 2022; 9:986943. 10.3389/fnut.2022.986943 36051905 PMC9424637

[18] Zhou YX, Xin HL, Rahman K, Wang SJ, Peng C, Zhang H. Portulaca oleracea L.: a review of phytochemistry and pharmacological effects. Biomed Res Int. 2015; 2015:925631. 10.1155/2015/925631 25692148 PMC4321094

[19] Farshori NN, Al-Sheddi ES, Al-Oqail MM, Musarrat J, Al-Khedhairy AA, Siddiqui MA. Cytotoxicity assessments of Portulaca oleracea and Petroselinum sativum seed extracts on human hepatocellular carcinoma cells (HepG2). Asian Pac J Cancer Prev. 2014; 15:6633–8. 10.7314/apjcp.2014.15.16.6633 25169500

[20] Asnani GP, Kokare CR. In vitro and in vivo evaluation of colon cancer targeted epichlorohydrin crosslinked Portulaca-alginate beads. Biomol Concepts. 2018; 9:190–9. 10.1515/bmc-2018-0019 30676996

[21] Alipour S, Pishkar L, Chaleshi V. Cytotoxic Effect of Portulaca Oleracea Extract on the Regulation of CDK1 and P53 Gene Expression in Pancreatic Cancer Cell Line. Nutr Cancer. 2022; 74:1792–801. 10.1080/01635581.2021.1960386 34431425

[22] Zhou X, Li Y, Li T, Cao J, Guan Z, Xu T, Jia G, Ma G, Zhao R. *Portulaca oleracea* L. Polysaccharide Inhibits Porcine Rotavirus In Vitro. Animals (Basel). 2023; 13:2306. 10.3390/ani13142306 37508085 PMC10376577

[23] Zhao R, Zhang T, Ma B, Li X. Antitumor Activity of Portulaca Oleracea L. Polysaccharide on HeLa Cells Through Inducing TLR4/NF-κB Signaling. Nutr Cancer. 2017; 69:131–9. 10.1080/01635581.2017.1248294 27911090

[24] Zhao R, Gao X, Cai Y, Shao X, Jia G, Huang Y, Qin X, Wang J, Zheng X. Antitumor activity of Portulaca oleracea L. polysaccharides against cervical carcinoma in vitro and in vivo. Carbohydr Polym. 2013; 96:376–83. 10.1016/j.carbpol.2013.04.023 23768576

[25] Shen H, Tang G, Zeng G, Yang Y, Cai X, Li D, Liu H, Zhou N. Purification and characterization of an antitumor polysaccharide from Portulaca oleracea L. Carbohydr Polym. 2013; 93:395–400. 10.1016/j.carbpol.2012.11.107 23499074

[26] Armstrong DK, Alvarez RD, Backes FJ, Bakkum-Gamez JN, Barroilhet L, Behbakht K, Berchuck A, Chen LM, Chitiyo VC, Cristea M, DeRosa M, Eisenhauer EL, Gershenson DM, et al. NCCN Guidelines® Insights: Ovarian Cancer, Version 3.2022. J Natl Compr Canc Netw. 2022; 20:972–80. 10.6004/jnccn.2022.0047 36075393

[27] An Y, Yang Q. Tumor-associated macrophage-targeted therapeutics in ovarian cancer. Int J Cancer. 2021; 149:21–30. 10.1002/ijc.33408 33231290

[28] O'Malley DM. New Therapies for Ovarian Cancer. J Natl Compr Canc Netw. 2019; 17:619–21. 10.6004/jnccn.2019.5018 31117037

[29] Chandra A, Pius C, Nabeel M, Nair M, Vishwanatha JK, Ahmad S, Basha R. Ovarian cancer: Current status and strategies for improving therapeutic outcomes. Cancer Med. 2019; 8:7018–31. 10.1002/cam4.2560 PMC6853829 31560828

[30] Fu Q, Zhou S, Yu M, Lu Y, He G, Huang X, Huang Y. Portulaca oleracea *Polysaccharides Modulate* Intestinal Microflora in Aged Rats *in vitro*. Front Microbiol. 2022; 13:841397. 10.3389/fmicb.2022.841397 35308364 PMC8931684

[31] Yi S, Jin X, Liu B, Wu P, Xiao W, Chen W. Portulaca oleracea extract reduces gut microbiota imbalance and inhibits colorectal cancer progression via inactivation of the Wnt/β-catenin signaling pathway. Phytomedicine. 2022; 105:154279. 10.1016/j.phymed.2022.154279 35963192

[32] Chen T, Wang J, Li Y, Shen J, Zhao T, Zhang H. Sulfated modification and cytotoxicity of Portulaca oleracea L. polysaccharides. Glycoconj J. 2010; 27:635–42. 10.1007/s10719-010-9307-0 20820911

[33] Lei G, Zhuang L, Gan B. Targeting ferroptosis as a vulnerability in cancer. Nat Rev Cancer. 2022; 22:381–96. 10.1038/s41568-022-00459-0 PMC10243716 35338310

[34] Mou Y, Wang J, Wu J, He D, Zhang C, Duan C, Li B. Ferroptosis, a new form of cell death: opportunities and challenges in cancer. J Hematol Oncol. 2019; 12:34. 10.1186/s13045-019-0720-y 30925886 PMC6441206

[35] Cheng J, Fan YQ, Liu BH, Zhou H, Wang JM, Chen QX. ACSL4 suppresses glioma cells proliferation via activating ferroptosis. Oncol Rep. 2020; 43:147–58. 10.3892/or.2019.7419 PMC6912066 31789401

[36] Yuan H, Li X, Zhang X, Kang R, Tang D. Identification of ACSL4 as a biomarker and contributor of ferroptosis. Biochem Biophys Res Commun. 2016; 478:1338–43. 10.1016/j.bbrc.2016.08.124 27565726

[37] Doll S, Proneth B, Tyurina YY, Panzilius E, Kobayashi S, Ingold I, Irmler M, Beckers J, Aichler M, Walch A, Prokisch H, Trümbach D, Mao G, et al. ACSL4 dictates ferroptosis sensitivity by shaping cellular lipid composition. Nat Chem Biol. 2017; 13:91–8. 10.1038/nchembio.2239 27842070 PMC5610546

[38] Wang Y, Zhang M, Bi R, Su Y, Quan F, Lin Y, Yue C, Cui X, Zhao Q, Liu S, Yang Y, Zhang D, Cao Q, Gao X. ACSL4 deficiency confers protection against ferroptosis-mediated acute kidney injury. Redox Biol. 2022; 51:102262. 10.1016/j.redox.2022.102262 35180475 PMC8857079

[39] Sha W, Hu F, Xi Y, Chu Y, Bu S. Mechanism of Ferroptosis and Its Role in Type 2 Diabetes Mellitus. J Diabetes Res. 2021; 2021:9999612. 10.1155/2021/9999612 34258295 PMC8257355

[40] Ji Q, Fu S, Zuo H, Huang Y, Chu L, Zhu Y, Hu J, Wu Y, Chen S, Wang Y, Ren Y, Pu X, Liu N, et al. ACSL4 is essential for radiation-induced intestinal injury by initiating ferroptosis. Cell Death Discov. 2022; 8:332. 10.1038/s41420-022-01127-w 35869042 PMC9307849

[41] Liu J, Kang R, Tang D. Signaling pathways and defense mechanisms of ferroptosis. FEBS J. 2022; 289:7038–50. 10.1111/febs.16059 34092035

[42] Ortega MA, Fraile-Martinez O, García-Montero C, Funes Moñux RM, Rodriguez-Martín S, Bravo C, De Leon-Luis JA, Saz JV, Saez MA, Guijarro LG, Lahera G, Mora F, Fernandez-Rojo S, et al. The Placentas of Women Who Suffer an Episode of Psychosis during Pregnancy Have Increased Lipid Peroxidation with Evidence of Ferroptosis. Biomolecules. 2023; 13:120. 10.3390/biom13010120 36671505 PMC9855415

[43] Cai W, Liu L, Shi X, Liu Y, Wang J, Fang X, Chen Z, Ai D, Zhu Y, Zhang X. Alox15/15-HpETE Aggravates Myocardial Ischemia-Reperfusion Injury by Promoting Cardiomyocyte Ferroptosis. Circulation. 2023; 147:1444–60. 10.1161/CIRCULATIONAHA.122.060257 36987924

